# Supplementary material for: HIVprotI: an integrated web based platform for prediction and design of HIV proteins inhibitors
Source: J Cheminform. 2018 Mar 9;10:12. doi: 10.1186/s13321-018-0266-y (PMC5845081; doi:10.1186/s13321-018-0266-y)
Supplement: Supplementary file 2 — Additional file 2. Source code of HIVProtI web server. [file 13321_2018_266_MOESM2_ESM.zip › HIVprotI_Source-code/hivprotI/weka-3-6-6/doc/weka/classifiers/bayes/net/search/SearchAlgorithm.html]

SearchAlgorithm


---


|  |  |  |
| --- | --- | --- |
| **Overview** \| **Package** \| **Class** \| **Tree** \| **Deprecated** \| **Index** \| **Help** \| **Weka's home** | |  |
| PREV CLASS   NEXT CLASS | **FRAMES**    **NO FRAMES**     **All Classes** |  |
| SUMMARY: NESTED \| FIELD \| CONSTR \| METHOD | DETAIL: FIELD \| CONSTR \| METHOD |  |


---


## weka.classifiers.bayes.net.search Class SearchAlgorithm

```
java.lang.Object
  weka.classifiers.bayes.net.search.SearchAlgorithm
```

**All Implemented Interfaces:**: java.io.Serializable, OptionHandler, RevisionHandler

**Direct Known Subclasses:**: FromFile, GlobalScoreSearchAlgorithm, LocalScoreSearchAlgorithm, NaiveBayes

---

``` public class SearchAlgorithm extends java.lang.Object implements OptionHandler, java.io.Serializable, RevisionHandler ```

This is the base class for all search algorithms for learning Bayes networks.
It contains some common code, used by other network structure search algorithms,
and should not be used by itself.


**Version:**
:   $Revision: 1.9 $

**Author:**
:   Remco Bouckaert

**See Also:**: Serialized Form

---

**Constructor Summary**

`SearchAlgorithm()`
  
          c'tor


| **Method Summary** | |
| --- | --- |
| `void` | `buildStructure(BayesNet bayesNet, Instances instances)`             buildStructure determines the network structure/graph of the network. |
| `java.lang.String[]` | `getOptions()`             Gets the current settings of the Classifier. |
| `java.lang.String` | `getRevision()`             Returns the revision string. |
| `java.lang.String` | `initAsNaiveBayesTipText()` |
| `java.util.Enumeration` | `listOptions()`             Returns an enumeration describing the available options. |
| `java.lang.String` | `maxNrOfParentsTipText()` |
| `void` | `setOptions(java.lang.String[] options)`             Parses a given list of options. |
| `java.lang.String` | `toString()`             a string representation of the algorithm |

**Methods inherited from class java.lang.Object**

`equals, getClass, hashCode, notify, notifyAll, wait, wait, wait`

**Constructor Detail**

### SearchAlgorithm

```
public SearchAlgorithm()
```

:   c'tor


**Method Detail**

### listOptions

```
public java.util.Enumeration listOptions()
```

:   Returns an enumeration describing the available options.

    :   **Specified by:**: `listOptions` in interface `OptionHandler`
    :   **Returns:**: an enumeration of all the available options.

---


### setOptions

```
public void setOptions(java.lang.String[] options)
                throws java.lang.Exception
```

:   Parses a given list of options.

    :   **Specified by:**: `setOptions` in interface `OptionHandler`
    :   **Parameters:**: `options` - the list of options as an array of strings **Throws:**: `java.lang.Exception` - if an option is not supported

---


### getOptions

```
public java.lang.String[] getOptions()
```

:   Gets the current settings of the Classifier.

    :   **Specified by:**: `getOptions` in interface `OptionHandler`
    :   **Returns:**: an array of strings suitable for passing to setOptions

---


### toString

```
public java.lang.String toString()
```

:   a string representation of the algorithm

    :   **Overrides:**: `toString` in class `java.lang.Object`
    :   **Returns:**: a string representation

---


### buildStructure

```
public void buildStructure(BayesNet bayesNet,
                           Instances instances)
                    throws java.lang.Exception
```

:   buildStructure determines the network structure/graph of the network.
    The default behavior is creating a network where all nodes have the first
    node as its parent (i.e., a BayesNet that behaves like a naive Bayes classifier).
    This method can be overridden by derived classes to restrict the class
    of network structures that are acceptable.

    :   **Parameters:**: `bayesNet` - the network: `instances` - the data to use **Throws:**: `java.lang.Exception` - if something goes wrong

---


### maxNrOfParentsTipText

```
public java.lang.String maxNrOfParentsTipText()
```

:   **Returns:**: a string to describe the MaxNrOfParentsoption.

---


### initAsNaiveBayesTipText

```
public java.lang.String initAsNaiveBayesTipText()
```

:   **Returns:**: a string to describe the InitAsNaiveBayes option.

---


### getRevision

```
public java.lang.String getRevision()
```

:   Returns the revision string.

    :   **Specified by:**: `getRevision` in interface `RevisionHandler`
    :   **Returns:**: the revision


---


|  |  |  |
| --- | --- | --- |
| **Overview** \| **Package** \| **Class** \| **Tree** \| **Deprecated** \| **Index** \| **Help** \| **Weka's home** | |  |
| PREV CLASS   NEXT CLASS | **FRAMES**    **NO FRAMES**     **All Classes** |  |
| SUMMARY: NESTED \| FIELD \| CONSTR \| METHOD | DETAIL: FIELD \| CONSTR \| METHOD |  |


---
